# (*E*)-1-(2-Hy­droxy-4,6-di­meth­oxy­phen­yl)-3-(naph­thalen-1-yl)prop-2-en-1-one

**DOI:** 10.1107/S2414314622009324

**Published:** 2022-09-27

**Authors:** Dongsoo Koh

**Affiliations:** a Department of Applied Chemistry, Dongduk Women’s University, Seoul, 02748, Republic of Korea; Sunway University, Malaysia

**Keywords:** crystal structure, chalcone, O—H⋯O hydrogen bond, C—H⋯O inter­actions

## Abstract

In the title compound, C_21_H_18_O_4_, the relative conformation of the C=C and C=O double bonds in the central enone group is *s-cisoid*; there is a *trans* configuration about the C=C bond. The dihedral angle formed by the naphthalene ring system and the benzene ring is 16.80 (2)°. The meth­oxy groups at the *ortho* and *para* positions of the benzene ring are tilted to the ring by 169.8 (1) and 174.5 (1)°, respectively. The hy­droxy group in the benzene ring participates in an intra­molecular O—H⋯O hydrogen bond. In the crystal, C—H⋯O inter­actions link mol­ecules into linear chains along the *a*-axis direction.

## Abstract 2

The crystal structure of a chalcone is reported. The relative conformation of the C=C and C=O double bonds in the central enone group is *s-cisoid*; there is a *trans* configuration about the C=C bond. In the crystal, C—H⋯O inter­actions link mol­ecules into linear chains along the *a*-axis direction.

## Graphical abstract

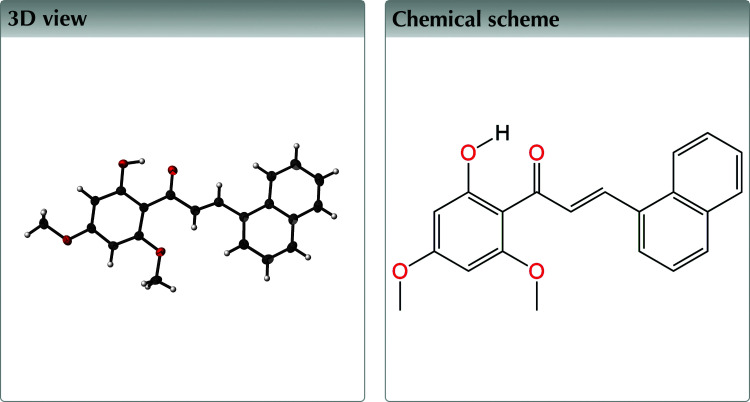

## Structure description

Reactive oxygen species (ROS) damage DNA, RNA, and proteins when present in excess. There is growing evidence that flavonoids can suppress carcinogenesis by inhibiting ROS levels (Rodríguez-García *et al.*, 2019 ▸). Surprisingly, flavonoids can also induce excessive oxidative stress, leading to cancer cell death (Slika *et al.*, 2022 ▸). Flavones (Hostetler *et al.*, 2017 ▸), aurones (Sui *et al.*, 2021 ▸), and chalcones (Elkanzi *et al.*, 2022 ▸), which belong to the sub-group of flavonoids, have in common an α,β-unsaturated carbonyl group in the mol­ecule. The α,β-unsaturated carbonyl group reacts with the thiol group of gluta­thione (GSH) as a Michael acceptor to reduce the intra­cellular GSH concentration (Adams *et al.*, 2012 ▸). Since cancer cells have a higher ROS concentration than normal cells (Kumari *et al.*, 2018 ▸), α,β-unsaturated carbonyl groups rapidly increase ROS levels due to decreased GSH, thereby killing cancer cells (Raj *et al.*, 2011 ▸). As an extension of the search for ROS-generating compounds in cancer cells (Shin *et al.*, 2022 ▸; Lee *et al.*, 2016 ▸), the title chalcone compound was synthesized.

**Figure 1:**
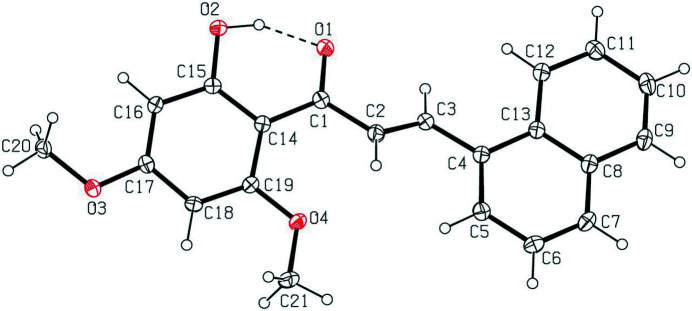
The mol­ecular structure of the title compound, showing the atom-labeling scheme and displacement ellipsoids drawn at the 50% probability level. The dashed bond represents the intra­molecular O—H⋯O hydrogen bond.

**Figure 2:**
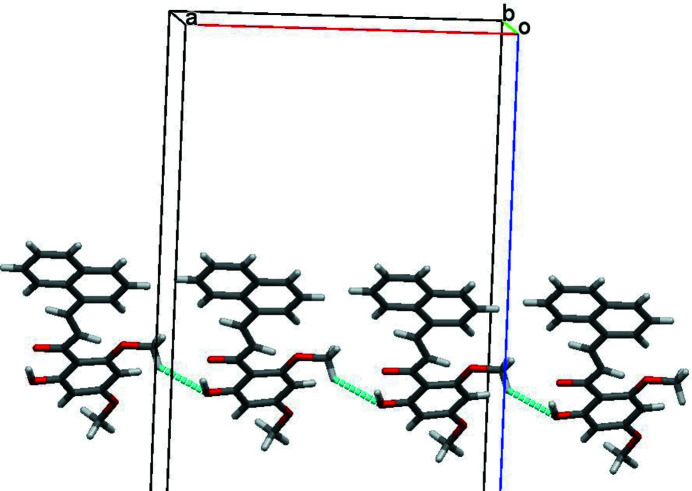
Part of the crystal structure of the title compound with weak inter­molecular C—H⋯O inter­actions shown as green dashed lines.

The mol­ecular structure of the title compound is shown in Fig. 1 ▸. In the central α,β-unsaturated carbonyl group, the carbonyl O1=C1 and C2=C3 double bonds are twisted at an angle of −22.9 (2)° for the C3—C2—C1—O1 torsion angle. A *trans-*configuration is noted for the C2=C3 double bond, which has a torsion angle of −178.3 (1)° for C1—C2—C3—C4. The meth­oxy group at the *para* position (C-17) of the benzene ring is nearly coplanar with the ring [C18—C17—O3—C20 = 174.5 (1)°], while the other meth­oxy group at the *ortho* position (C-19) is more twisted out of the ring [C14—C19—O4—C21 = 169.8 (1)°]. The naphthalene ring system (C4–C13; r.m.s. deviation of 0.003 Å) is tilted at an angle of 16.80 (2)° with respect to the benzene ring (C14–C19; r.m.s. deviation of 0.011 Å). The hy­droxy group attached to the benzene ring is involved in an intra­molecular O—H⋯O hydrogen bond. In the crystal, weak C—H⋯O inter­actions link the mol­ecules into linear chains propagating along the *a*-axis direction (Fig. 2 ▸, Table 1 ▸).

## Synthesis and crystallization

1-(2-Hy­droxy-4,6-di­meth­oxy­phen­yl)ethanone (196 mg, 1 mmol) and 1-naphthaldehyde (156 mg, 1 mmol) were dissolved in ethanol (25 ml) and the temperature was cooled to around 276–277 K in an ice bath. To the cooled reaction mixture were added 1.0 ml of 40% aqueous KOH solution, and the reaction mixture was stirred at room temperature for 20 h. This mixture was poured into iced water (40 ml) and acidified with 6 *N* HCl solution. The mixture was extracted with ethyl acetate (2 × 30 ml) and the combined organic layers were dried over MgSO_4_. Filtration and evaporation of the filtrate gave a residue which was purified by flash chromatography to give the title compound (260 mg, 78%). Recrystallization in ethanol gave the crystals used in this X-ray diffraction study.

## Refinement

Crystal data, data collection and structure refinement details are summarized in Table 2 ▸.

## Supplementary Material

Crystal structure: contains datablock(s) I. DOI: 10.1107/S2414314622009324/tk4085sup1.cif

Structure factors: contains datablock(s) I. DOI: 10.1107/S2414314622009324/tk4085Isup2.hkl

Supporting information file. DOI: 10.1107/S2414314622009324/tk4085Isup3.cml

CCDC reference: 2208753

Additional supporting information:  crystallographic information; 3D view; checkCIF report

## Figures and Tables

**Table 1:** Hydrogen-bond geometry (Å, °)

*D*—H⋯*A*	*D*—H	H⋯*A*	*D*⋯*A*	*D*—H⋯*A*
O2—H2*O*⋯O1	0.94 (2)	1.60 (2)	2.4869 (12)	155.5 (18)
C21—H21*C*⋯O2^i^	0.98	2.58	3.3393 (17)	135

Symmetry code: (i) 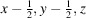 .

**Table 2:** Experimental details

Crystal data	
Chemical formula	C_21_H_18_O_4_
*M*_r_	334.35
Crystal system, space group	Monoclinic, *C*2/*c*
Temperature (K)	147
*a*, *b*, *c* (Å)	15.8594 (7), 5.0437 (2), 40.6908 (17)
β (°)	90.507 (2)
*V* (Å^3^)	3254.7 (2)
*Z*	8
Radiation type	Cu *K*α
μ (mm^−1^)	0.77
Crystal size (mm)	0.65 × 0.11 × 0.03

Data collection	
Diffractometer	Bruker Kappa *APEX* DUO CCD
Absorption correction	Multi-scan (*SADABS*; Bruker, 2012 ▸)
*T*_min_, *T*_max_	0.655, 0.753
No. of measured, independent and observed [*I* > 2σ(*I*)] reflections	10590, 2743, 2555
*R*_int_	0.027
(sin θ/λ)_max_ (Å^−1^)	0.595

Refinement	
*R*[*F* ^2^ > 2σ(*F* ^2^)], *wR*(*F* ^2^), *S*	0.037, 0.098, 1.03
No. of reflections	2743
No. of parameters	232
H-atom treatment	H atoms treated by a mixture of independent and constrained refinement
Δρ_max_, Δρ_min_ (e Å^−3^)	0.16, −0.22

Computer programs: *APEX2* and *SAINT* (Bruker, 2012 ▸), *SHELXS* and *SHELXTL* (Sheldrick, 2008 ▸), *SHELXL2014* (Sheldrick, 2015 ▸) and *publCIF* (Westrip, 2010 ▸).

## full crystallographic data

### Crystal data

**Table:**

C_21_H_18_O_4_	*F*(000) = 1408
*M**_r_* = 334.35	*D*_x_ = 1.365 Mg m^−^^3^
Monoclinic, *C*2/*c*	Cu *K*α radiation, λ = 1.54178 Å
Hall symbol: -C 2yc	Cell parameters from 6156 reflections
*a* = 15.8594 (7) Å	θ = 6.5–66.5°
*b* = 5.0437 (2) Å	µ = 0.77 mm^−^^1^
*c* = 40.6908 (17) Å	*T* = 147 K
β = 90.507 (2)°	Needle, yellow
*V* = 3254.7 (2) Å^3^	0.65 × 0.11 × 0.03 mm
*Z* = 8	

### Data collection

**Table:**

Bruker Kappa APEX DUO CCD diffractometer	2743 independent reflections
Radiation source: Bruker ImuS	2555 reflections with *I* > 2σ(*I*)
Multi-layer optics monochromator	*R*_int_ = 0.027
φ and ω scans	θ_max_ = 66.5°, θ_min_ = 6.5°
Absorption correction: multi-scan (SADABS; Bruker, 2012)	*h* = −18→18
*T*_min_ = 0.655, *T*_max_ = 0.753	*k* = −5→1
10590 measured reflections	*l* = −48→47

### Refinement

**Table:**

Refinement on *F*^2^	Primary atom site location: structure-invariant direct methods
Least-squares matrix: full	Secondary atom site location: difference Fourier map
*R*[*F*^2^ > 2σ(*F*^2^)] = 0.037	Hydrogen site location: inferred from neighbouring sites
*wR*(*F*^2^) = 0.098	H atoms treated by a mixture of independent and constrained refinement
*S* = 1.03	*w* = 1/[σ^2^(*F*_o_^2^) + (0.0514*P*)^2^ + 2.058*P*] where *P* = (*F*_o_^2^ + 2*F*_c_^2^)/3
2743 reflections	(Δ/σ)_max_ = 0.001
232 parameters	Δρ_max_ = 0.16 e Å^−^^3^
0 restraints	Δρ_min_ = −0.22 e Å^−^^3^

### Special details

**Table:**

Geometry. All esds (except the esd in the dihedral angle between two l.s. planes) are estimated using the full covariance matrix. The cell esds are taken into account individually in the estimation of esds in distances, angles and torsion angles; correlations between esds in cell parameters are only used when they are defined by crystal symmetry. An approximate (isotropic) treatment of cell esds is used for estimating esds involving l.s. planes.
Refinement. Refinement of F^2^ against ALL reflections. The weighted R-factor wR and goodness of fit S are based on F^2^, conventional R-factors R are based on F, with F set to zero for negative F^2^. The threshold expression of F^2^ > 2sigma(F^2^) is used only for calculating R-factors(gt) etc. and is not relevant to the choice of reflections for refinement. R-factors based on F^2^ are statistically about twice as large as those based on F, and R- factors based on ALL data will be even larger.

### Fractional atomic coordinates and isotropic or equivalent isotropic displacement parameters (Å^2^)

**Table:**

	*x*	*y*	*z*	*U*_iso_*/*U*_eq_	
O1	0.83835 (5)	0.92735 (19)	0.40390 (2)	0.0331 (2)	
O2	0.82944 (5)	1.26680 (18)	0.44807 (2)	0.0286 (2)	
O3	0.56231 (5)	1.62372 (18)	0.47470 (2)	0.0289 (2)	
O4	0.57849 (5)	0.93109 (17)	0.39751 (2)	0.0277 (2)	
C1	0.75984 (8)	0.9014 (2)	0.40286 (3)	0.0233 (3)	
C2	0.72497 (8)	0.6836 (3)	0.38301 (3)	0.0278 (3)	
H2A	0.6702	0.6195	0.3880	0.033*	
C3	0.76623 (8)	0.5731 (2)	0.35859 (3)	0.0267 (3)	
H3A	0.8215	0.6365	0.3543	0.032*	
C4	0.73370 (8)	0.3599 (2)	0.33742 (3)	0.0239 (3)	
C5	0.64822 (8)	0.3106 (3)	0.33554 (3)	0.0284 (3)	
H5A	0.6109	0.4154	0.3483	0.034*	
C6	0.61469 (9)	0.1099 (3)	0.31536 (3)	0.0309 (3)	
H6A	0.5555	0.0802	0.3147	0.037*	
C7	0.66664 (9)	−0.0425 (3)	0.29668 (3)	0.0294 (3)	
H7A	0.6435	−0.1779	0.2831	0.035*	
C8	0.75456 (8)	−0.0002 (2)	0.29737 (3)	0.0250 (3)	
C9	0.80929 (9)	−0.1570 (3)	0.27802 (3)	0.0319 (3)	
H9A	0.7863	−0.2930	0.2645	0.038*	
C10	0.89410 (10)	−0.1162 (3)	0.27848 (3)	0.0371 (3)	
H10A	0.9298	−0.2226	0.2653	0.045*	
C11	0.92890 (9)	0.0833 (3)	0.29851 (4)	0.0363 (3)	
H11A	0.9882	0.1111	0.2988	0.044*	
C12	0.87801 (8)	0.2379 (3)	0.31759 (3)	0.0294 (3)	
H12A	0.9026	0.3716	0.3310	0.035*	
C13	0.78924 (8)	0.2026 (2)	0.31770 (3)	0.0232 (3)	
C14	0.70640 (8)	1.0824 (2)	0.42166 (3)	0.0212 (3)	
C15	0.74516 (7)	1.2624 (2)	0.44389 (3)	0.0220 (3)	
C16	0.69958 (8)	1.4415 (2)	0.46266 (3)	0.0230 (3)	
H16A	0.7273	1.5536	0.4781	0.028*	
C17	0.61307 (8)	1.4532 (2)	0.45848 (3)	0.0232 (3)	
C18	0.57152 (8)	1.2849 (2)	0.43638 (3)	0.0240 (3)	
H18A	0.5121	1.2976	0.4335	0.029*	
C19	0.61656 (8)	1.1008 (2)	0.41876 (3)	0.0216 (3)	
C20	0.60019 (9)	1.7843 (3)	0.49980 (3)	0.0298 (3)	
H20A	0.5563	1.8845	0.5112	0.045*	
H20B	0.6402	1.9079	0.4898	0.045*	
H20C	0.6300	1.6705	0.5156	0.045*	
C21	0.48877 (9)	0.9150 (3)	0.39821 (4)	0.0405 (4)	
H21A	0.4699	0.7638	0.3849	0.061*	
H21B	0.4645	1.0787	0.3893	0.061*	
H21C	0.4703	0.8914	0.4209	0.061*	
H2O	0.8485 (12)	1.142 (4)	0.4325 (5)	0.064 (6)*	

### Atomic displacement parameters (Å^2^)

**Table:**

	*U*^11^	*U*^22^	*U*^33^	*U*^12^	*U*^13^	*U*^23^
O1	0.0226 (5)	0.0357 (5)	0.0409 (5)	0.0010 (4)	0.0022 (4)	−0.0128 (4)
O2	0.0200 (5)	0.0321 (5)	0.0336 (5)	−0.0005 (4)	−0.0009 (4)	−0.0081 (4)
O3	0.0249 (5)	0.0318 (5)	0.0300 (5)	0.0043 (4)	0.0027 (4)	−0.0074 (4)
O4	0.0236 (5)	0.0297 (5)	0.0297 (5)	−0.0045 (4)	−0.0016 (4)	−0.0066 (4)
C1	0.0257 (6)	0.0215 (6)	0.0226 (6)	−0.0003 (5)	0.0024 (5)	0.0037 (5)
C2	0.0258 (7)	0.0260 (7)	0.0318 (7)	−0.0011 (5)	0.0024 (5)	−0.0035 (5)
C3	0.0247 (6)	0.0260 (7)	0.0294 (6)	0.0011 (5)	−0.0008 (5)	−0.0029 (5)
C4	0.0280 (7)	0.0229 (6)	0.0208 (6)	0.0006 (5)	−0.0006 (5)	0.0020 (5)
C5	0.0289 (7)	0.0280 (7)	0.0283 (6)	0.0008 (5)	0.0018 (5)	−0.0009 (5)
C6	0.0278 (7)	0.0334 (7)	0.0313 (7)	−0.0060 (6)	−0.0026 (5)	0.0016 (6)
C7	0.0385 (8)	0.0259 (7)	0.0238 (6)	−0.0061 (6)	−0.0059 (5)	−0.0001 (5)
C8	0.0352 (7)	0.0216 (6)	0.0182 (6)	0.0002 (5)	−0.0021 (5)	0.0035 (5)
C9	0.0446 (8)	0.0268 (7)	0.0243 (6)	0.0008 (6)	−0.0007 (6)	−0.0043 (5)
C10	0.0420 (8)	0.0352 (8)	0.0342 (7)	0.0078 (6)	0.0074 (6)	−0.0073 (6)
C11	0.0303 (7)	0.0374 (8)	0.0414 (8)	0.0035 (6)	0.0042 (6)	−0.0048 (6)
C12	0.0298 (7)	0.0275 (7)	0.0310 (7)	0.0000 (5)	−0.0001 (5)	−0.0041 (5)
C13	0.0293 (7)	0.0209 (6)	0.0194 (6)	0.0013 (5)	−0.0011 (5)	0.0030 (5)
C14	0.0236 (6)	0.0195 (6)	0.0206 (6)	−0.0016 (5)	0.0017 (5)	0.0024 (5)
C15	0.0215 (6)	0.0224 (6)	0.0219 (6)	−0.0017 (5)	0.0004 (5)	0.0040 (5)
C16	0.0257 (6)	0.0225 (6)	0.0208 (6)	−0.0020 (5)	0.0002 (5)	−0.0015 (5)
C17	0.0261 (6)	0.0220 (6)	0.0217 (6)	0.0018 (5)	0.0044 (5)	0.0019 (5)
C18	0.0200 (6)	0.0264 (7)	0.0257 (6)	−0.0005 (5)	0.0007 (5)	0.0020 (5)
C19	0.0243 (6)	0.0210 (6)	0.0195 (6)	−0.0037 (5)	0.0003 (5)	0.0026 (5)
C20	0.0331 (7)	0.0314 (7)	0.0249 (6)	0.0060 (6)	0.0009 (5)	−0.0055 (5)
C21	0.0231 (7)	0.0492 (9)	0.0490 (9)	−0.0054 (6)	−0.0050 (6)	−0.0144 (7)

### Geometric parameters (Å, º)

**Table:**

O1—C1	1.2523 (15)	C9—C10	1.361 (2)
O2—C15	1.3461 (15)	C9—H9A	0.9500
O2—H2O	0.94 (2)	C10—C11	1.405 (2)
O3—C17	1.3538 (15)	C10—H10A	0.9500
O3—C20	1.4316 (15)	C11—C12	1.3688 (19)
O4—C19	1.3549 (15)	C11—H11A	0.9500
O4—C21	1.4258 (16)	C12—C13	1.4190 (18)
C1—C14	1.4656 (17)	C12—H12A	0.9500
C1—C2	1.4688 (18)	C14—C15	1.4181 (17)
C2—C3	1.3182 (19)	C14—C19	1.4316 (17)
C2—H2A	0.9500	C15—C16	1.3896 (17)
C3—C4	1.4685 (18)	C16—C17	1.3823 (17)
C3—H3A	0.9500	C16—H16A	0.9500
C4—C5	1.3797 (18)	C17—C18	1.3974 (18)
C4—C13	1.4360 (17)	C18—C19	1.3769 (17)
C5—C6	1.4051 (19)	C18—H18A	0.9500
C5—H5A	0.9500	C20—H20A	0.9800
C6—C7	1.363 (2)	C20—H20B	0.9800
C6—H6A	0.9500	C20—H20C	0.9800
C7—C8	1.4105 (19)	C21—H21A	0.9800
C7—H7A	0.9500	C21—H21B	0.9800
C8—C9	1.4178 (18)	C21—H21C	0.9800
C8—C13	1.4232 (18)		
			
C15—O2—H2O	103.2 (12)	C11—C12—C13	121.35 (12)
C17—O3—C20	117.37 (10)	C11—C12—H12A	119.3
C19—O4—C21	117.53 (10)	C13—C12—H12A	119.3
O1—C1—C14	119.78 (11)	C12—C13—C8	117.82 (11)
O1—C1—C2	117.78 (11)	C12—C13—C4	123.08 (11)
C14—C1—C2	122.42 (11)	C8—C13—C4	119.09 (11)
C3—C2—C1	122.95 (12)	C15—C14—C19	115.88 (11)
C3—C2—H2A	118.5	C15—C14—C1	118.83 (11)
C1—C2—H2A	118.5	C19—C14—C1	125.25 (11)
C2—C3—C4	125.29 (12)	O2—C15—C16	116.16 (11)
C2—C3—H3A	117.4	O2—C15—C14	121.02 (11)
C4—C3—H3A	117.4	C16—C15—C14	122.82 (11)
C5—C4—C13	118.47 (11)	C17—C16—C15	118.74 (11)
C5—C4—C3	120.29 (11)	C17—C16—H16A	120.6
C13—C4—C3	121.22 (11)	C15—C16—H16A	120.6
C4—C5—C6	121.93 (12)	O3—C17—C16	124.13 (11)
C4—C5—H5A	119.0	O3—C17—C18	114.87 (11)
C6—C5—H5A	119.0	C16—C17—C18	121.00 (11)
C7—C6—C5	120.30 (12)	C19—C18—C17	120.06 (11)
C7—C6—H6A	119.8	C19—C18—H18A	120.0
C5—C6—H6A	119.8	C17—C18—H18A	120.0
C6—C7—C8	120.41 (12)	O4—C19—C18	121.88 (11)
C6—C7—H7A	119.8	O4—C19—C14	116.69 (10)
C8—C7—H7A	119.8	C18—C19—C14	121.43 (11)
C7—C8—C9	120.98 (12)	O3—C20—H20A	109.5
C7—C8—C13	119.80 (11)	O3—C20—H20B	109.5
C9—C8—C13	119.22 (12)	H20A—C20—H20B	109.5
C10—C9—C8	121.20 (12)	O3—C20—H20C	109.5
C10—C9—H9A	119.4	H20A—C20—H20C	109.5
C8—C9—H9A	119.4	H20B—C20—H20C	109.5
C9—C10—C11	119.97 (13)	O4—C21—H21A	109.5
C9—C10—H10A	120.0	O4—C21—H21B	109.5
C11—C10—H10A	120.0	H21A—C21—H21B	109.5
C12—C11—C10	120.43 (13)	O4—C21—H21C	109.5
C12—C11—H11A	119.8	H21A—C21—H21C	109.5
C10—C11—H11A	119.8	H21B—C21—H21C	109.5
			
O1—C1—C2—C3	−22.91 (19)	C3—C4—C13—C8	−179.47 (11)
C14—C1—C2—C3	158.34 (12)	O1—C1—C14—C15	−7.79 (17)
C1—C2—C3—C4	−178.35 (11)	C2—C1—C14—C15	170.93 (11)
C2—C3—C4—C5	18.8 (2)	O1—C1—C14—C19	169.92 (11)
C2—C3—C4—C13	−162.71 (13)	C2—C1—C14—C19	−11.36 (18)
C13—C4—C5—C6	0.90 (18)	C19—C14—C15—O2	−178.57 (10)
C3—C4—C5—C6	179.46 (12)	C1—C14—C15—O2	−0.65 (16)
C4—C5—C6—C7	−0.4 (2)	C19—C14—C15—C16	1.93 (17)
C5—C6—C7—C8	−0.08 (19)	C1—C14—C15—C16	179.84 (11)
C6—C7—C8—C9	−179.90 (12)	O2—C15—C16—C17	177.50 (10)
C6—C7—C8—C13	0.03 (18)	C14—C15—C16—C17	−2.97 (18)
C7—C8—C9—C10	179.69 (12)	C20—O3—C17—C16	−5.82 (17)
C13—C8—C9—C10	−0.25 (19)	C20—O3—C17—C18	174.47 (10)
C8—C9—C10—C11	0.3 (2)	C15—C16—C17—O3	−178.27 (11)
C9—C10—C11—C12	−0.1 (2)	C15—C16—C17—C18	1.42 (17)
C10—C11—C12—C13	−0.2 (2)	O3—C17—C18—C19	−179.20 (10)
C11—C12—C13—C8	0.25 (19)	C16—C17—C18—C19	1.08 (18)
C11—C12—C13—C4	179.78 (12)	C21—O4—C19—C18	−11.21 (17)
C7—C8—C13—C12	−179.98 (11)	C21—O4—C19—C14	169.78 (11)
C9—C8—C13—C12	−0.05 (17)	C17—C18—C19—O4	178.90 (10)
C7—C8—C13—C4	0.46 (17)	C17—C18—C19—C14	−2.14 (18)
C9—C8—C13—C4	−179.60 (11)	C15—C14—C19—O4	179.68 (10)
C5—C4—C13—C12	179.56 (11)	C1—C14—C19—O4	1.91 (17)
C3—C4—C13—C12	1.01 (18)	C15—C14—C19—C18	0.67 (16)
C5—C4—C13—C8	−0.91 (17)	C1—C14—C19—C18	−177.10 (11)

### Hydrogen-bond geometry (Å, º)

**Table:**

*D*—H···*A*	*D*—H	H···*A*	*D*···*A*	*D*—H···*A*
O2—H2*O*···O1	0.94 (2)	1.60 (2)	2.4869 (12)	155.5 (18)
C21—H21*C*···O2^i^	0.98	2.58	3.3393 (17)	135

Symmetry code: (i) *x*−1/2, *y*−1/2, *z*.
